# Structure and heme-binding properties of HemQ (chlorite dismutase-like protein) from *Listeria monocytogenes*

**DOI:** 10.1016/j.abb.2015.01.010

**Published:** 2015-05-15

**Authors:** Stefan Hofbauer, Andreas Hagmüller, Irene Schaffner, Georg Mlynek, Michael Krutzler, Gerhard Stadlmayr, Katharina F. Pirker, Christian Obinger, Holger Daims, Kristina Djinović-Carugo, Paul G. Furtmüller

**Affiliations:** a Department of Chemistry, Division of Biochemistry, BOKU – University of Natural Resources and Life Sciences, Muthgasse 18, A-1190 Vienna, Austria; b Department for Structural and Computational Biology, Max F. Perutz Laboratories, University of Vienna, A-1030 Vienna, Austria; c Department of Biochemistry, Faculty of Chemistry and Chemical Technology, University of Ljubljana, 1000 Ljubljana, Slovenia; d Department of Microbiology and Ecosystem Science, Division of Microbial Ecology, University of Vienna, A-1090 Vienna, Austria

**Keywords:** Chlorite dismutase, HemQ, Heme binding, Heme biosynthesis, Protein stability, X-ray crystallography

## Abstract

Chlorite dismutase-like proteins are structurally closely related to functional chlorite dismutases which are heme *b*-dependent oxidoreductases capable of reducing chlorite to chloride with simultaneous production of dioxygen. Chlorite dismutase-like proteins are incapable of performing this reaction and their biological role is still under discussion. Recently, members of this large protein family were shown to be involved in heme biosynthesis in Gram-positive bacteria, and thus the protein was renamed HemQ in these organisms. In the present work the structural and heme binding properties of the chlorite dismutase-like protein from the Gram-positive pathogen *Listeria monocytogenes* (LmCld) were analyzed in order to evaluate its potential role as a regulatory heme sensing protein. The homopentameric crystal structure (2.0 Å) shows high similarity to chlorite-degrading chlorite dismutases with an important difference in the structure of the putative substrate and heme entrance channel. In solution LmCld is a stable hexamer able to bind the low-spin ligand cyanide. Heme binding is reversible with *K*_D_-values determined to be 7.2 μM (circular dichroism spectroscopy) and 16.8 μM (isothermal titration calorimetry) at pH 7.0. Both acidic and alkaline conditions promote heme release. Presented biochemical and structural data reveal that the chlorite dismutase-like protein from *L. monocytogenes* could act as a potential regulatory heme sensing and storage protein within heme biosynthesis.

## Highlights

• The crystal structure of apo-LmCld is presented.
• LmCld binds heme reversibly and cooperatively.
• LmCld is equipped to act as HemQ in the heme biosynthesis of *L. monocytogenes.*

## Graphical abstract

**Figure:**
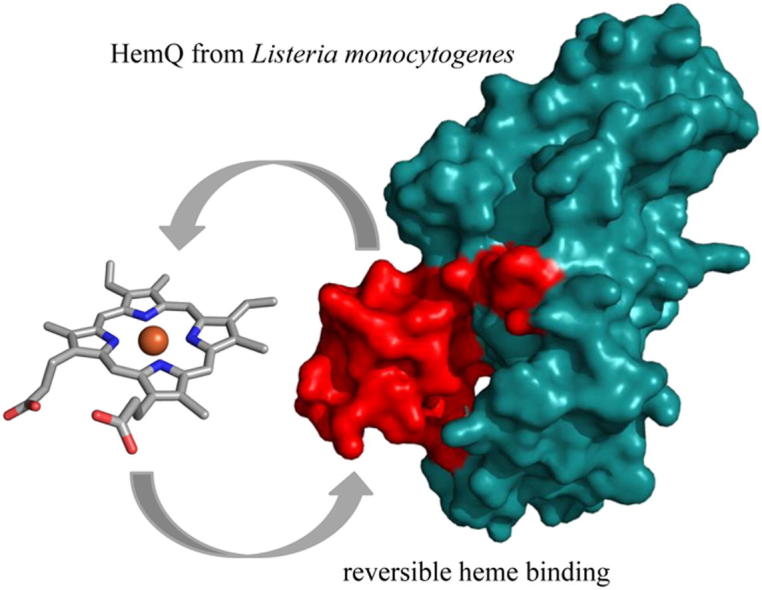

## Introduction

The Gram-positive bacterium *Listeria monocytogenes* is a ubiquitous pathogen that thrives in diverse environments such as soil, water, various food products, humans, and animals. The disease caused by this bacterium, listeriosis, is acquired by ingesting contaminated food products and mainly affects immunocompromised individuals, pregnant women, new-born children, and elderly people. Listeriosis manifests as gastroenteritis, meningitis, encephalitis and septicaemia, resulting in death in 25–30% of cases [1].

Current progress in genetics, fed by the burst in genome sequence data, has led to the identification of novel bacterial heme proteins that are now characterized in structural and mechanistic terms. One of those is the chlorite dismutase-like (Cld-like)^1^ protein from *L. monocytogenes* (LmCld). Phylogenetic studies showed that LmCld shares a common ancestor with dye-decolorizing peroxidases (DyP) and the canonical chlorite dismutases (Clds) [2,3]. Dye-decolorizing peroxidases, originally found to oxidize anthraquinone dyes from which the denomination dye-decolorizing peroxidase was derived [4], play an important role in lignin degradation [5], but they also oxidize small synthetic peroxidase substrates like guaiacol and ABTS. The physiological role of canonical chlorite dismutases in perchlorate reducing bacteria is to degrade chlorite to harmless chloride and dioxygen, but their roles in other phyla as nitrite-oxidizing bacteria are unknown [3,6,7]. Besides the phylogenetic data, little is known about the physiological role of Cld-like proteins. The Cld-like protein from *Haloferax volcanii* was hypothesized to be relevant in antibiotic biosynthesis. Together with a monooxygenase-like protein, its gene is located within a single open reading frame [8]. Cld-like proteins from *Bacillus subtilis*, *Mycobacterium tuberculosis* [9], and *Staphylococcus aureus* [10] have been reported to play a (yet undefined) role in heme biosynthesis. First implications that Cld-like proteins are involved in heme biosynthesis came from analysis of available genomes of Actinobacteria where the genes for Cld-like proteins are co-operonic with genes for the heme biosynthetic pathway [9]. The most striking observation is that in *Propionibacterium acnes* the Cld-like protein is fused to the *hemH* gene coding for the ferrochelatase (Fc) which is responsible for the last step of heme biosynthesis, the incorporation of the heme iron. Based on these findings the Cld-like protein was renamed into HemQ [9]. Prior to this work, Cld-like proteins (HemQs) from other Firmicutes, like *B. subtilis* [9] and *S. aureus* [10], were studied in detail. These studies show that *ΔhemQ* deficient strains of e.g. *S. aureus* exhibit a changed phenotype (small colony variants) and can be rescued by supplementation with hemin, which proves HemQ’s role in heme biosynthesis [10]. In Firmicutes genes for Cld-like proteins were not found in association with other genes encoding for heme biosynthesis proteins [9]. However, a gene for a Cld-like protein (HemQ) exists in all heme-synthesizing Gram-positive organisms, not just in the Actinobacteria [9,10]. Analysis of putative signal sequences revealed that all Cld-like proteins, including HemQs in Gram-positive bacteria, as well as all chlorite degrading Clds from Clade II are located in the cytosol, whereas functional Clds of Clade I are secreted to the periplasm [11].

In the protein data bank (www.rcsb.org) there are three structures from Cld-like proteins deposited, none of those have a heme *b* bound. While for the Cld-like protein from *Thermus thermophilus* (Deinococcus–Thermus) (pdb-code: 1VDH) heme binding was shown [12], no information besides the deposited structures are available for Cld-like proteins from archaeal species *Thermoplasma acidophilum* (pdb-code: 3DTZ) and for the Firmicutes species *Geobacillus stearothermophilus* (pdb-code: 1T0T). The amino acid residues around the potential heme *b* binding site in *L. monocytogenes* differ from the ones in other Cld-like proteins that can be found in the protein data bank [3] (Fig. S1).

In this study, we have addressed the question of whether the yet uncharacterized Cld-like protein from *L. monocytogenes* might bind and/or transfer heme. For this purpose, we expressed both the heme *b*-bound holo-protein and the heme-free apo-protein in *Escherichia coli*. A comprehensive study of catalytic and biochemical properties reveals a weak cooperative heme binding, conditionally stable protein with little or no catalytic activity toward small redox substrates but with the ability to transfer heme to other proteins. Based on the determined crystal structure of the apo-protein we discuss heme binding properties in relation to structurally related proteins from other bacteria including functional chlorite dismutases.

## Materials and methods

### Cloning, heterologous expression and purification

The coding sequence of *cld* homolog (ORF annotated as *lmo2113* in the EGD-e genome, accession no. NC003210) was PCR-amplified using genomic DNA of *L. monocytogenes* L028 as template and primers OSF17 and OSF18. After purification and digestion with *Nco*I (5′-CCATGG-3′) and *Xho*I (5′-CTCGAG-3′), the PCR fragment was subcloned into a modified version of pET21(+) expression vector with an N-terminal StrepII-tag, cleavable by TEV protease [StrepTEVpET21(+)]. Recombinant LmCld was expressed in *E. coli* Tuner (DE3) cells (Merck/Novagen, Darmstadt, Germany) which were grown in 2 L Erlenmeyer flasks in 500 mL Luria–Bertani (LB) medium. The LB medium was supplemented with ampicillin (100 μg mL^−1^) and was inoculated with a freshly prepared overnight culture. The culture was grown at 37 °C and 180 rpm agitation until OD_600_ was approximately 0.6. Prior to induction, the cultivation temperature was lowered to 24 °C and 50 μg mL^−1^ (final concentration) hemin was added. The culture was induced for expression with 0.5 mM (final concentration) isopropyl-β-d-thiogalactopyranoside (IPTG). After 4 h the culture was harvested by centrifugation (2430×*g*, 10 min). The resulting cell pellet was stored at −30 °C until further use. For purification the cell pellet was thawed and suspended in 50 mM HEPES, pH 7.4, 5% glycerol, 0.5% Triton X-100, 0.5 mM EDTA, and 1 mM phenylmethylsulfonylfluoride (PMSF). The cell suspension was lysed under sonication (Vibra Cell, Sonics & Materials Inc., Danbury, CT, USA; 50% power, 3 × 60 s) and clarified by centrifugation (38,720×*g*, 25 min, 4 °C). The supernatant was filtered (0.45 μm, Millipore) and loaded onto a StrepTrap HP 5 mL (GE Healthcare) column, equilibrated with 20 mM HEPES, pH 7.4, 2% glycerol. Elution was performed using 20 mM HEPES, pH 7.4, 2% glycerol, and 1 mM desthiobiotin. Obtained proteins were screened by SDS–PAGE and fractions containing LmCld were pooled and concentrated. For crystallization the StrepII-tag was fully cleaved off using TEV-protease in 20 mM HEPES, pH 7.4, 2% glycerol, and 0.5 mM DTT. Further, the sample was applied to a HiLoad 16/60 Superdex 200 pg (GE Healthcare) column, equilibrated with 20 mM HEPES, pH 7.4, 2% glycerol. TEV protease (27 kDa) was separated from LmCld (180 kDa) by size exclusion chromatography in this step. Prior to this, cleaved StrepII-tag and uncleaved protein were removed using a StrepTrap column. Aliquots of purified proteins were concentrated to 5 mg mL^−1^ and stored at −80 °C until further use.

### Determination of the oligomeric assembly in solution

Size-exclusion chromatography combined with multi-angle light scattering was performed to determine the molar mass and oligomeric state of apo-LmCld and holo-LmCld. HPLC (Shimadzu prominence LC20) was equipped with MALS (WYATT Heleos Dawn8+ plus QELS; software ASTRA 6), refractive index detector (RID-10A, Shimadzu) and a diode array detector (SPD-M20A, Shimadzu). The column (Superdex 200 10/300 GL, GE healthcare) had a particle size of 13 μm was equilibrated with Dulbecco PBS plus 200 mM NaCl. Experiments were performed at a flow rate of 0.75 mL per min and the injected protein amount was 50 μg. The proper performance of molar mass calculation by MALS was verified by the determination of a sample of bovine serum albumin.

### Crystallization

Pure recombinant apo-LmCld crystallized in optimized conditions of formulation 87 of the SaltRX screen (Hampton Research), 0.1 M Tris, pH 8.5, 0.6 M potassium sodium tartrate tetrahydrate with 8% (v/v) glycerol. For cryo-protection the glycerol concentration was further elevated to 25% (v/v) by adding mother liquor with glycerol to the drops. For crystallization in optimized conditions 24-well Linbro format hanging drop plates (Crystalgen) were used.

### Data collection, processing, phasing and refinement

Diffraction data from LmCld crystals were collected at synchrotron beamlines (ESRF ID14-1, ESRF ID 23-1). Integration and scaling was done with XDS and XSCALE [13]. The phases for all data sets were derived from molecular replacement by the online server BALBES [14]. Refinement of LmCld structures was carried out in Phenix Refine [15] and COOT [16].

### Substrate channel calculation

CAVER [17] was used to detect putative channels to the heme binding site of apo-LmCld (pdb-code: 4WWS) and NdCld (pdb-code: 3NN1). For calculation of the characteristics of the channels, the heme iron of NdCld was set as a starting point for both proteins, which were structurally aligned. Channels were calculated with the following settings: minimum probe radius: 0.9 Å; shell depth: 10 Å; shell radius: 9 Å; clustering threshold: 3.5; number of approximating balls: 12; input atoms: 20 amino acids (without other components present in the respective structural model).

### Titration of LmCld with hemin

Electronic circular dichroism (ECD) and absorption spectra were recorded (Chirascan, Applied Photophysics, Leatherhead, UK) between 260 and 500 nm (1 s nm^−1^ scan speed, 3 nm bandwidth, 10 mm pathlength). The instrument was flushed with a nitrogen flow of 5 L min^−1^ and allowed simultaneous monitoring of UV–vis electronic absorption and circular dichroism. Protein concentration of apo-LmCld was 40 μM in 50 mM phosphate buffer, pH 7.0. Hemin was added from a 1 mM stock solution (presolved in 0.5 M NaOH and diluted to working concentration with 100 mM phosphate buffer, pH 7.0). Resulting electronic absorption spectra were corrected for dilution and the excess of free hemin present in the solution. Free hemin in solution does not show any ellipticity in circularly polarized light due to the lack of a chiral center, therefore no correction of the ECD-spectra was necessary.

Isothermal titration calorimetry (ITC) was performed to determine binding constants for the binding of hemin to apo-LmCld. All titrations were performed in 50 mM phosphate buffer, pH 7.0. Hemin solutions were freshly prepared prior to each titration to minimize hemin aggregation. Hemin was dissolved in a minimal volume of 0.5 M NaOH, further diluted in 50 mM phosphate buffer, pH 7.0 and centrifuged at maximum rotations speed for 5 min. The concentration was measured using an extinction coefficient value of *ε*_385_ = 58,440 M^−1^ cm^−1^ [18]. ITC was performed with an ITC200 titration calorimeter (Microcal) with a cell volume of 200 μL. The maximum volume of the titrant was 40 μL. Typically, 500 μM hemin solution was added to 35 μM apo-LmCld as one 0.5 μL – followed by thirty-four 1 μL-injections, using a syringe rotating at 400 rpm. An interval of 3 min between successive injections was given to allow the baseline to stabilize. All titrations were performed at 25 °C.

Titration data points were fitted best with a sigmoidal curve (Hill equation), from which the binding constant (*K*_D_) and the Hill coefficient (*n*) were determined [19].

### UV–visible electronic absorption spectroscopy at various pH values

Spectra of 3 μM holo-LmCld were recorded on a UV–visible spectrophotometer (Hitachi U-3900) between 250 and 700 nm (300 nm min^−1^) in 50 mM citrate/phosphate buffer (pH 4.0–7.0), 50 mM phosphate buffer (pH 6.5–8.0), and glycine buffer (pH 7.5–10.0) in absence and presence of 150 mM NaCl.

### Secondary structure determination of LmCld at various pH values and temperature-mediated unfolding

Electronic circular dichroism (ECD) spectra of 12 μM LmCld were recorded (Chirascan, Applied Photophysics, Leatherhead, UK) in the far UV-region (190–260 nm) for secondary structure determination at pH 4.0 (5 mM citrate/phosphate buffer), pH 7.0 (5 mM phosphate buffer), and pH 10 (5 mM glycine buffer). The spectral bandwidth was set to 3 nm and the scan speed was 10 s nm^−1^, the pathlength was 1 mm. Spectra were recorded at 20 °C. The instrument was equipped with a Peltier element for temperature control and temperature mediated unfolding was monitored at 208 nm between 20 °C and 85 °C (final temperature in the cuvette). The temperature was increased continuously at a rate of 1 °C min^−1^. A wavelength scan of the sample was performed at 85 °C with the same settings as described above. The sample was cooled down to 20 °C and another wavelength scan was performed to test the degree of reversible unfolding of LmCld. Secondary structure calculations were carried out with CDNN (Applied Photophysics).

Temperature-mediated unfolding was additionally tested with differential scanning calorimetry (DSC) using a VP-capillary DSC microcalorimeter from Microcal (cell volume: 137 μL), controlled by the VP-viewer program and equipped with an autosampler for 96 well plates. Samples were analyzed within 20–120 °C with a heating rate of 60 °C h^−1^ at 60 psi (4.136 bar) cell pressure. Collected DSC data were corrected for buffer baseline and normalized for protein concentration. Conditions: 35 μM holo- and apo-LmCld in 50 mM phosphate buffer pH 7.0. For data analysis and conversion the Microcal origin software was used.

### Temperature-dependent heme binding

ECD spectra of 6 μM LmCld were recorded in the near UV- and visible region (260–500 nm; 1 s nm^−1^ scan speed, 3 nm bandwidth, 10 mm pathlength) (Chirascan, Applied Photophysics, Leatherhead, UK) at 20 °C, 90 °C and after cooling again at 20 °C (to demonstrate reversibility). The temperature-induced release of the heme co-factor was monitored by the loss of ellipticity at 417 nm. Temperature was continuously increased at a rate of 1 °C min^−1^.

Electronic absorption spectra of 4 μM LmCld were recorded at various temperatures between 250 and 700 nm on a UV–visible spectrophotometer (Hitachi U-3900) at a scan rate of 600 nm min^−1^. The cuvette-holder was equipped with a magnetic stirrer allowing constant mixing of the sample and a circulating water bath for temperature control. Temperature was increased stepwise and spectra were recorded at 25, 30, 40, 45, 50, 55, 60, 65, and 70 °C and afterwards the temperature was decreased stepwise and spectra were recorded at before mentioned temperature points. As soon as the target temperature was reached, the sample was equilibrated for at least 15 min at the desired temperature before the measurement was performed.

### Heme transfer

The transfer of the prosthetic group from holo-LmCld to excess apo-myoglobin (apo-Mb) was monitored photometrically by the increase of absorbance at 409 nm, similarly as reported previously [20]. Apo-myoglobin (from horse heart, Sigma) was prepared by using a modified extraction method by Teale [21]. In detail, 1.5 mL distilled ice-cold water were adjusted to pH 2.2 with HCl and 100 μL of ∼500 μM holo-Mb were added. Further, ice-cold 1.6 mL 2-Butanol (Merck) were added, vortexed for 30 s and left on ice until phases were completely separated. Apo-Mb was present in the lower aqueous phase and was pipetted into 5 mL 100 mM phosphate buffer, pH 7.0, for neutralisation. Apo-Mb was washed with 50 mL 100 mM phosphate buffer, pH 7.0, and concentrated in Amicon-Ultra centrifugal filters with a 10 kDa cut-off (Millipore). Heme transfer experiments were conducted on a UV–visible spectrophotometer (Hitachi U-3900) and in 100 mM phosphate buffer, pH 7.0, at 25 °C. Each measurement was started by the addition of 5, 7.5, 10, and 15 μM apo-Mb (final concentration) to 1 μM holo-LmCld (final concentration). Data was collected for 20 min and time traces were fitted by a double-exponential equation to obtain rate constants (*k*_obs1_, *k*_obs2_) for heme loss of holo-LmCld. Data were normalized by subtracting the baseline at 409 nm (autozero prior to measurement) to obtain Δ*A*_409_.

### Enzyme activity measurements and cyanide binding

Catalase activity was tested within hydrogen peroxide concentrations from 0 to 10 mM, chlorite degrading activity was tested with chlorite concentrations up to 1 mM in 50 mM phosphate buffer, pH 7.0. In both cases putative oxygen generation was followed using a Clark-type oxygen electrode (Oxygraph Plus, Hansatech Instruments, Norfolk, UK), inserted into a stirred water bath kept at 30 °C, the reaction volume was 1 mL. The electrode was equilibrated to 100% O_2_ saturation by bubbling with O_2_ through the reaction mixture and to 0% saturation by bubbling with N_2_ until plateaus were reached to derive an offset and calibration factor.

Cyanide binding experiments were conducted with a stopped-flow apparatus (model SX-18MV, Applied Photophysics) equipped for both conventional and sequential measurements. The optical quartz cell with a path length of 10 mm had a volume of 20 μL. The fastest time for mixing was 1 ms. All measurements were performed at 25 °C. For the cyanide binding studies with ferric holo-LmCld, the conventional stopped-flow mode was used and the decrease in absorbance at 412 nm was monitored. In a typical experiment 3 μM holo-LmCld in 100 mM phosphate buffer, pH 7.0 were mixed with at least 10-fold excess of cyanide in the same buffer. A minimum of three measurements were performed for each ligand concentration. Additionally, the binding of cyanide to holo-LmCld was investigated using the diode array detector (Applied Photophysics), which enables to acquire sets of time-resolved spectra from a single stopped-flow drive.

### Structural data

Coordinates and structure factors were deposited to the RCSB Protein Data Bank (www.pdb.org). The accession number is 4WWS.

## Results

### Expression, purification and oligomeric structure

**Fig. 1:**
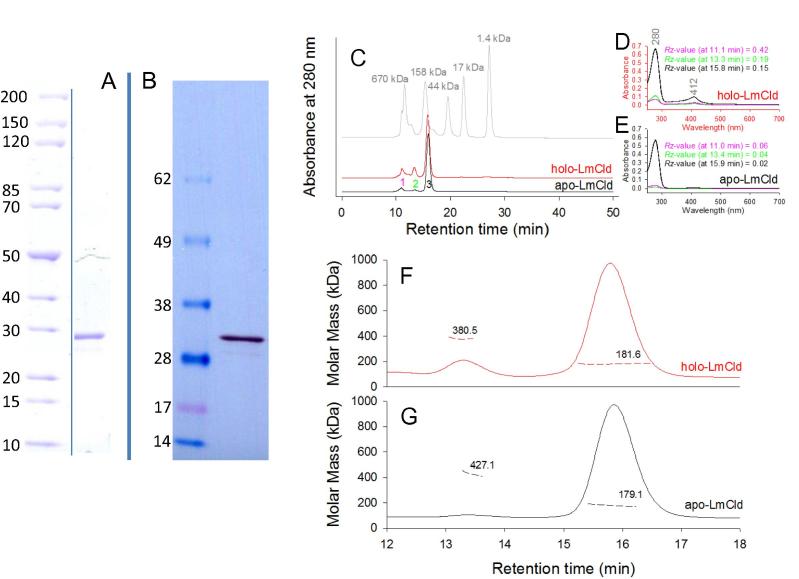
Subunit size and oligomeric structure in solution. SDS–PAGE (A), Western blot (B) of purified LmCld. (C) HPLC-chromatogram of holo-LmCld (red), apo-LmCld (black) and Bio-Rad Gel Filtration Standard (#151-1901) (gray), chromatograms are shifted for clarity. UV–vis spectra of peaks eluted at 11.0 (pink), 13.3 (green), and 15.9 min (black) of holo-LmCld (D) and apo-LmCld (E). Multi-angle-light-scattering (MALS) data of holo-LmCld (F) and apo-LmCld (G), molecular mass distributions over the elution peak of the samples are represented as dashed lines, average molar masses are written next to respective size distributions. (For interpretation of the references to color in this figure legend, the reader is referred to the web version of this article.)

Apo- and holo-LmCld were expressed in *E. coli* Tuner (DE3) cells and purified *via* StrepTrap affinity chromatography with a yield of approximately 5 mg of pure protein per liter of culture. The purified enzyme exhibits a subunit size of approximately 30 kDa (Fig. 1A and B). In size-exclusion chromatography, the majority of apo-LmCld and holo-LmCld elutes after 15.9 min (Fig. 1C) and light scattering data clearly suggest a hexameric assembly, corresponding to a molecular weight of approximately 180 kDa. In apo-LmCld and in holo-LmCld two minor fractions elute earlier after 13.3 min and 11.1 min (breakthrough of void volume), respectively. Light scattering data of the first fraction did not give reliable results, but an approximation suggests that around 24 subunits (4 × 6-mers) are assembled. The second fraction (around 13.3 min) is most probably arranged of 12 subunits (2 × 6-mers) (Fig. 1F and G) and both of the first two minor fractions are present in a higher percentage in holo-LmCld than in apo-LmCld (Fig. 1C–E). It has to be mentioned that heme is lost over time, especially if holo-LmCld is exposed to high salt concentrations as present in the running buffer that was required for HPLC measurements (340 mM NaCl), as will be described below (Fig. 4A, inset). This explains the low *Reinheitszahl* (*R*_Z_) (*A*_Soret_/*A*_280_) in these measurements. Interestingly, the *R*_Z_-value is higher when holo-LmCld is present as 12-mer or at even higher oligomeric assemblies.

### Structure

**Fig. 2:**
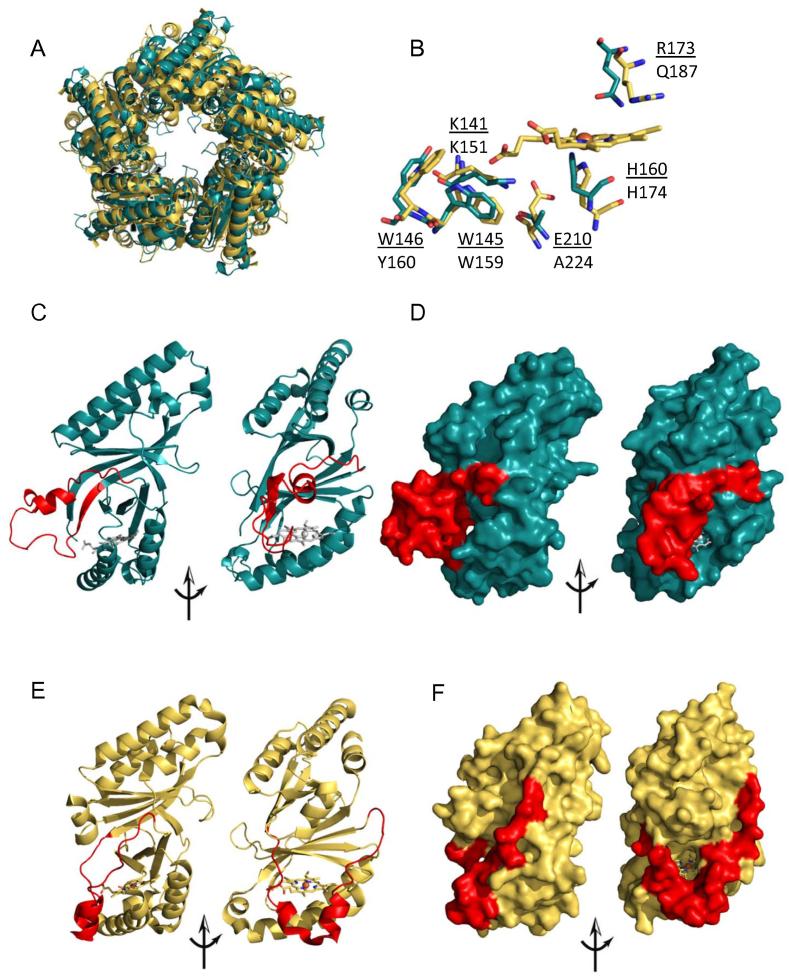
Crystal structure of apo-LmCld. (A) Overlay of pentameric apo-LmCld (green) and pentameric holo-NdCld (pdb-code: 3NN1) (yellow). (B) Overlay of the heme-binding sites of apo-LmCld (green) and holo-NdCld (yellow); residue-labels of holo-NdCld are underlined. Ribbon representation of apo-LmCld (C) and holo-NdCld (E); for better orientation the heme group of holo-NdCld was placed to the heme binding site of apo-LmCld (gray). Representation of the surfaces of apo-LmCld (D) and holo-NdCld (F). Substrate-channel forming secondary structural elements of holo-NdCld and respective residues of apo-LmCld are depicted in red (C–F). Figures were generated using PyMOL (http://www.pymol.org/). (For interpretation of the references to color in this figure legend, the reader is referred to the web version of this article.)

A crystal structure of apo-LmCld was solved and refined at a resolution of 2.0 Å (Table 1). The overall structure of apo-LmCld subunit is composed of an N- and a C-terminal ferredoxin-like domain, as also observed in other Clds. LmCld subunit is structurally similar to chlorite dismutases from “*Candidatus* Nitrospira defluvii” (pdb-code: 3NN1) [22], *Dechloromonas aromatica* (pdb-code: 3Q08) [23], and Cld-like protein from *T. thermophilus* (pdb-code: 1VDH) [12] [r.m.s.d. 1.714 Å (4WWS to 3NN1)]. In the crystal lattice apo-LmCld forms a homopentamer (Fig. 2A), compared to the hexameric assembly in solution. No electron density corresponding to heme was detected in the active site cavity of the C-terminal ferredoxin-like domain, despite the presence of a potential histidine residue as proximal ligand. In functional Clds a hydrogen bond between the proximal histidine and a neighboring glutamate stabilizes the position of the heme ligand. In contrast, an alanine replaces the glutamate in apo-LmCld. The distal site differs in having a glutamine residue at the position of the distal arginine which was shown to be of high importance for catalytic chlorite degrading activity (Fig. 2B) [22,24–27]. Moreover, similar to the Cld-like proteins from *T. thermophilus* and *G. stearothermophilus*, apo-LmCld contains a flexible stretch of amino acids between residues V105 and K141, as indicated by higher temperature factors and weaker electron density for these residues (Fig. 2C–D). This flexible stretch of residues aligns with the substrate channel forming α-helix of chlorite degrading chlorite dismutases (Fig. 2E and F) and has a major impact on the access to the heme binding site, as shown by tunnel calculations in the respective proteins using CAVER [17] (Fig. S2). While in NdCld the access to the heme is very large, in apo-LmCld the access is significantly different and narrower, since the flexible stretch of amino acids is positioned in front of the putative heme binding site (Fig. 2C and D), as also reflected in diverse characteristics of the access channels. The two most important channels in LmCld are given by throughput-values (throughput-values range from 0 to 1; the higher the value, the greater the importance of the pathway) of 0.81 (for both), bottleneck radii of 2.2 and 2.1 Å, and lengths of 14.5 and 13.9 Å. For the two most important tunnels in NdCld, characteristics reveal throughput-values of 0.89 and 0.86, bottleneck radii of 3.2 and 2.6, and lengths of 11.2 Å, respectively.

### Heme binding

**Fig. 3:**
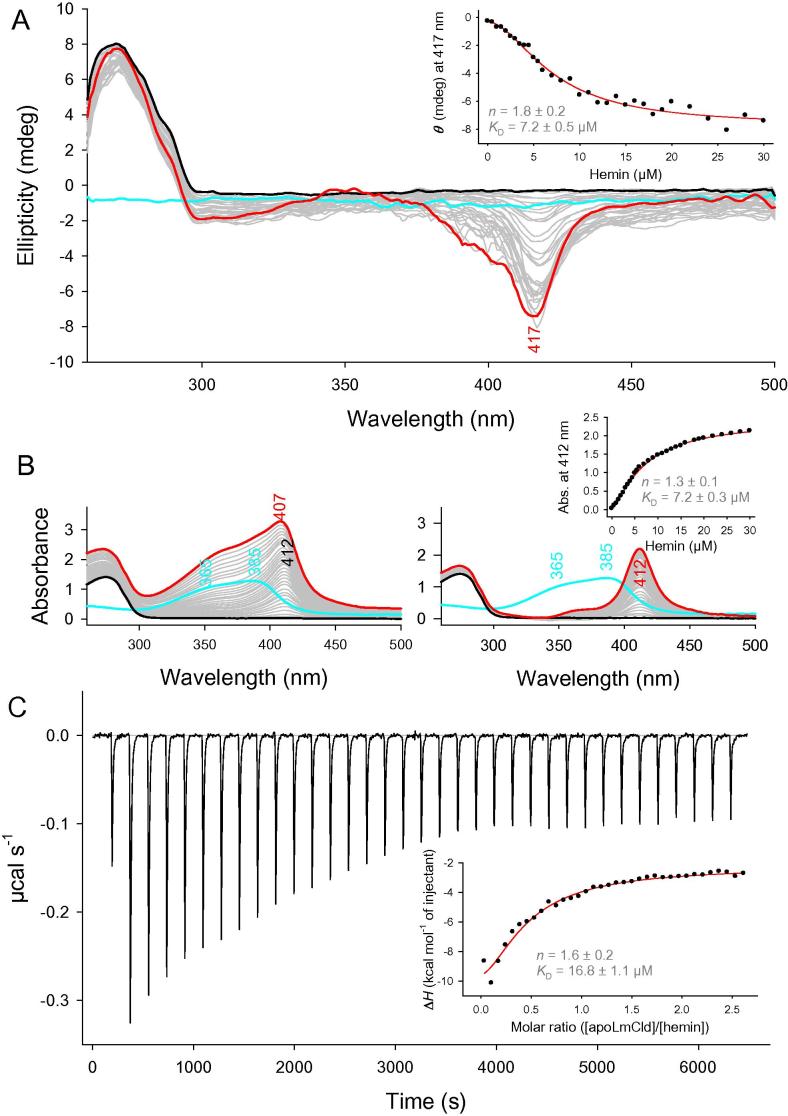
Hemin binding to apo-LmCld. Hemin binding followed spectroscopically by ECD and UV–vis (A and B) and calorimetrically by ITC (C). 35 μM apo-LmCld were titrated with hemin up to a molar ratio of 1.0 (A and B) and 2.5 (C) in 100 mM phosphate buffer, pH 7.0. (A) represents ECD spectra, the starting spectrum is shown in black, the resulting spectrum in red, intermediate spectra in gray, free hemin is depicted in cyan, the inset shows intensities at the ECD absorption minimum of (black dots); the sigmoidal fit is depicted as a red line. (B) shows UV–vis absorption spectra of the same experiment as in (A); spectra on the left are uncorrected measured spectra, spectra on the right side are corrected for excess hemin; the inset shows intensities at the absorption maximum (black dots) and a sigmoidal fit (red line). (C) depicts exotherms derived from each injection of the titrant, hemin, to the protein; the inset shows the plot of the integrated peak areas from the exotherms (black dots) with a sigmoidal fit (red line). (For interpretation of the references to color in this figure legend, the reader is referred to the web version of this article.)

The ability of apo-LmCld to bind heme was determined by titration of hemin to the apo-protein. This titration was followed spectroscopically (ECD, UV–vis) and calorimetrically (ITC). Free hemin has a UV–vis absorption spectrum with maxima at 365 and 385 nm. Hemin binds specifically to the protein and the resulting UV–vis spectrum is comprised of the sum of reconstituted LmCld with a Soret maximum at 412 nm and free hemin in solution. The amount of free hemin in the solution can be subtracted to yield the holo-LmCld spectrum with 50% heme occupancy (Fig. 3B). To overcome the interference of free hemin in solution, we followed specific heme binding with ECD spectroscopy in the visible range, since free hemin in solution is lacking any chirality and therefore does not give any ellipticity (Fig. 3A). Holo-LmCld shows an ellipticity minimum in the Soret region at 417 nm. Binding of hemin to apo-LmCld was monitored by the decrease of ellipticity at the Soret minimum.

Binding of hemin to apo-LmCld was also analyzed using isothermal titration calorimetry. Fig. 3C shows a titration of hemin to apo-LmCld. Due to unspecific binding of hemin to saturated LmCld observed in ITC experiments, Δ*H* of each injection does not become zero, as it was also observed for the heme binding of apo-forms of dye-decolorizing peroxidases from *Bacteroides thetaiotaomicron* and *Shewanella oneidensis* [28].

Titration data from ITC and ECD could be fitted best with a sigmoidal curve (insets to Fig. 3), yielding Hill-coefficients (*n*) of 1.6 ± 0.2 (ITC) and 1.8 ± 0.2 (ECD), which are indicative of a cooperative binding process. Half-maximal heme-binding occurred at molar ratios ([hemin]/[apo-LmCld]) of 0.48 ± 0.04 (ITC) and of 0.29 ± 0.02 (ECD) and calculated *K*_D_-values of 16.8 ± 1.1 μM (ITC) and 7.2 ± 0.5 μM (ECD).

### Influence of pH on heme binding

**Fig. 4:**
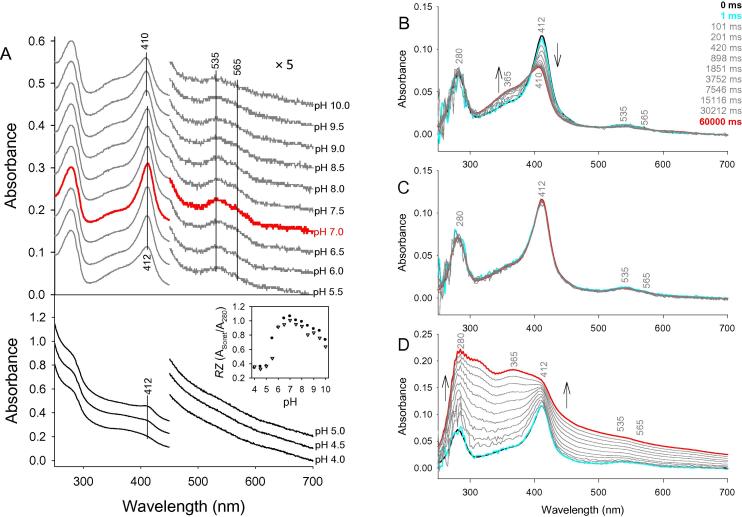
Effect of pH on heme binding to apo-LmCld. (A) shows 3 μM holo-LmCld at different pH values; conditions: 50 mM citrate/phosphate buffer, pH 4.0–7.0; 50 mM phosphate buffer, pH 6.5–8.0; 50 mM glycine buffer, pH 7.5–10.0. The inset depicts the pH dependence of the *Reinheitszahl* (*R*_Z_) of holo-LmCld; black dots are in absence of and gray triangles in presence of 150 mM NaCl. In (B)–(D) 4 μM holo LmCld were present in 5 mM phosphate buffer pH 7.0 and diluted rapidly using stopped-flow technique with 100 mM citrate/phosphate buffer, pH 4.0 (B), 100 mM phosphate buffer, pH 7.0 (C), and 100 mM glycine buffer, pH 10.0 (D). The starting spectra are depicted in black, spectra after 1 ms in cyan and resulting spectra in red, intermediate spectra are represented in gray. (For interpretation of the references to color in this figure legend, the reader is referred to the web version of this article.)

Since no crystal structure of holo-LmCld is available, holo-LmCld was studied spectroscopically over a wide pH range to investigate the status of the bound heme. Heme binding of holo-LmCld exhibits a pH dependence and has its optimum at pH 7.0, represented by the highest *R*_Z_-value (Fig. 4A). At acidic conditions the protein precipitates and loses its Soret band absorbance, indicating the release of the prosthetic group by the concomitant formation of a new maximum at 365 nm. In presence of 150 mM NaCl, *R*_Z_-values decrease further and the acidic transition appears at even higher pH (Fig. 4A, inset). At alkaline conditions the *R*_Z_ values decrease slightly and absorbance at 365 nm is increasing, the Soret maximum is 2 nm blue-shifted above pH 9.0. In pH-jump experiments (Fig. 4B–D) the same spectral transitions are observed at pH 4.0 and pH 10.0 within 60 s.

### Thermal stability of LmCld

**Fig. 5:**
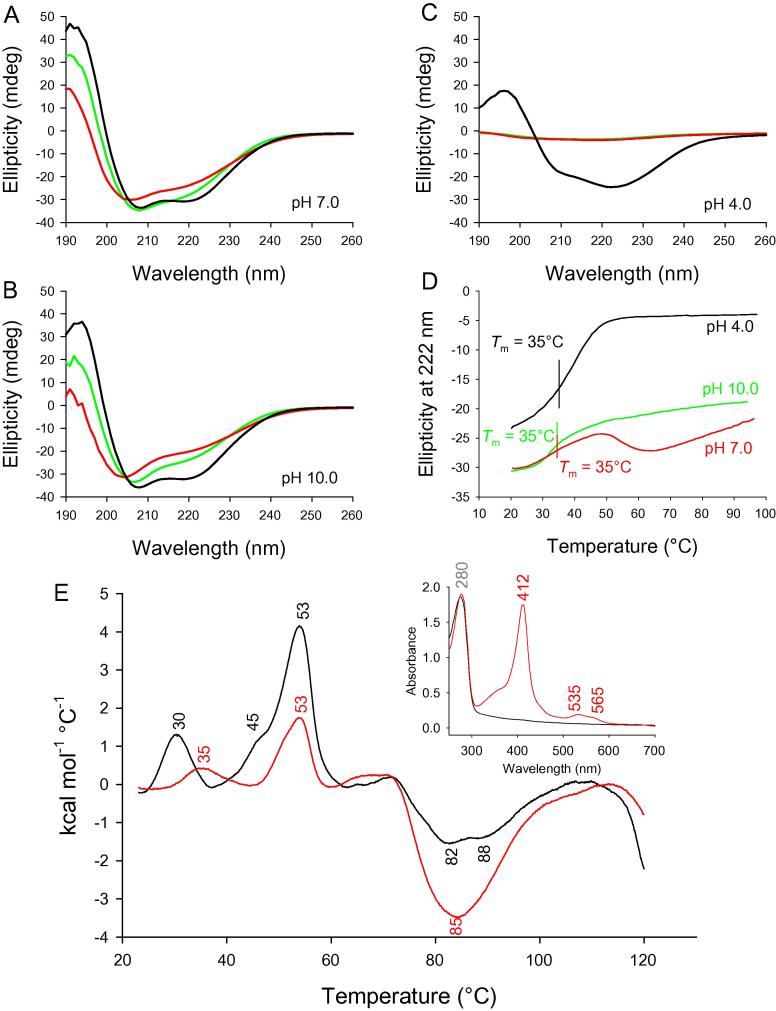
Thermal stability of LmCld. ECD spectra of 12 μM holo-LmCld at (A) pH 7.0 (50 mM phosphate buffer), (B) pH 10.0 (50 mM glycine buffer), and (C) pH 4.0 (50 mM citrate/phosphate buffer), at 20 °C (black lines), after heating to 85 °C (red lines), and after cooling again to 20 °C (green lines). (D) Temperature mediated unfolding of 12 μM holo-LmCld followed at 222 nm at pH 4.0 (black), pH 7.0 (red), and pH 10.0. (E) DSC thermograms of 35 μM apo-LmCld (black) and 35 μM holo-LmCld (red) in 50 mM phosphate buffer, pH 7.0. The inset depicts UV–vis spectra of samples used in the DSC experiment; apo-LmCld (black), holo-LmCld (red). (For interpretation of the references to color in this figure legend, the reader is referred to the web version of this article.)

In order to understand the heme-binding properties of LmCld, we investigated its secondary structure and its temperature dependence. Holo-LmCld in its stable fold (at pH 7.0–10.0) is comprised of equal amounts of α-helices and β-sheets or turns (Fig. 5). At acidic pH (4.0) more than half of the overall ellipticity is lost due to acidic denaturation (Fig. 5C), with the residual ellipticity being comprised of a higher content of α-helices and a smaller amount of antiparallel β-sheet structures. Upon heating, holo-LmCld at pH 4.0 is completely denatured with a broad transition (*T*_m_ = 35 °C) that is completed at approximately 50 °C, exhibiting complete loss of any ellipticity. After cooling, the sample remained unaltered and did not show any ellipticity, clearly suggesting irreversible unfolding (Fig. 5C). At pH 7.0 and 10.0 ellipticity changes at 222 nm upon heating are more difficult to interpret but also show a transition midpoint at approximately 35 °C. Holo-LmCld at pH 7.0 shows more transitions above 50 °C (Fig. 5D). In contrast to acidic conditions, significant ellipticity remained at 85 °C at neutral (65%) and alkaline (47%) pH (Fig. 5A and B). After cooling to 20 °C partial refolding occurs and overall ellipticity is regained to a certain extent (pH 7.0: 90%; pH 10.0: 73%).

Furthermore, differential scanning calorimetry (DSC) was performed to test the overall thermal stability of holo- and apo-LmCld. DSC thermograms exhibit several transitions (Fig. 5E). At low temperatures an endotherm at 30 °C (apo-LmCld) and 35 °C (holo-LmCld) occurs that resembles structural rearrangement observed in thermo-ECD measurements. Another prominent transition for both proteins has its midpoint at 53 °C. This transition can be assigned to a structural rearrangement responsible for the release of the heme co-factor (see below) that is more pronounced in the apo-protein, indicating a lower overall thermal and conformational stability due to the larger endotherm. Consequently, a correct incorporation of the heme is beneficial for LmCld’s stability. Apo-LmCld and holo-LmCld both exhibit large and broad exotherms at temperatures higher than 70 °C, indicating aggregation.

### Reversibility of heme binding and dissociation

**Fig. 6:**
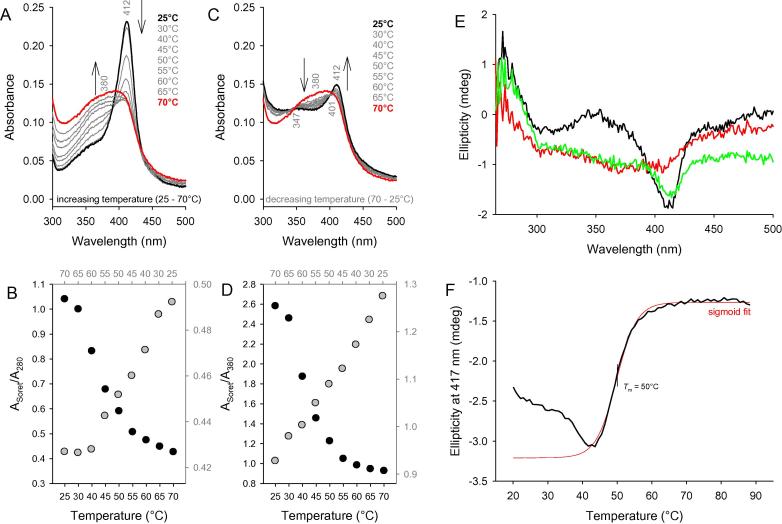
Reversibility of heme binding and dissociation. UV–vis spectral transitions when heating (A) from 25 °C and 70 °C and cooling from 70 °C to 25 °C (C) of 4 μM holo-LmCld. Plot of the *Reinheitzahl* (B) and *A*_Soret_/*A*_380_ ratio (D) during heating (black dots and labels) and cooling (gray dots and labels). (E) ECD spectra of 6 μM holo-LmCld at 20 °C (black), 90 °C (red), and 20 °C after cooling (green); (F) Temperature mediated release of the prosthetic group followed at the Soret minimum at 414 nm (black line) with its sigmoid fit (red line). Conditions: 100 mM phosphate buffer, pH 7.0. (For interpretation of the references to color in this figure legend, the reader is referred to the web version of this article.)

Temperature dependence of heme-binding of holo-LmCld was investigated with UV–vis absorption and ECD spectroscopy. In UV–vis absorption spectroscopy a loss of absorbance of the Soret band (412 nm) is observed with a simultaneous rise in absorbance at 380 nm, representing an increasing amount of free heme in solution (Fig. 6A). The overall *R*_Z_*-*value decreases with rising temperatures (Fig. 6B, black dots) as well as the ratio (*A*_Soret_/*A*_380_) between free and bound heme (Fig. 6D, black dots). Upon cooling the same sample, both *R*_Z_*-*values and *A*_Soret_/*A*_380_ ratios rise again due to an increase and sharpening of the Soret band at 412 nm (Fig. 6C), without regaining its initial value (Fig. 6B and D, gray dots). Plots of the *Reinheitzahl* (*A*_Soret_/*A*_280_) and the ratio of free heme to bound heme (*A*_Soret_/*A*_380_) from heating and cooling experiments reveal line-crossings between 45 °C and 50 °C, which are a good estimate for the release of the heme co-factor of holo-LmCld.

ECD spectroscopy in the near UV- and visible region of holo-LmCld reveals an intensity minimum of the Soret band at 417 nm (Fig. 6E, black line). Active chlorite dismutases from “*Candidatus* Nitrospira defluvii” and *Nitrobacter winogradskyi* show an ellipticity maximum in the Soret region [29]. The loss of ellipticity in the Soret region was followed at 417 nm and revealed a midpoint transition at 50 °C. A spectrum of holo-LmCld recorded at 90 °C shows no ellipticity in the Soret region (Fig. 6E, red line), but after cooling the sample to 20 °C, a certain percentage of ellipticity of the Soret band is regained, indicating rebinding of the heme to the protein (Fig. 6E, green line).

### Heme transfer

**Fig. 7:**
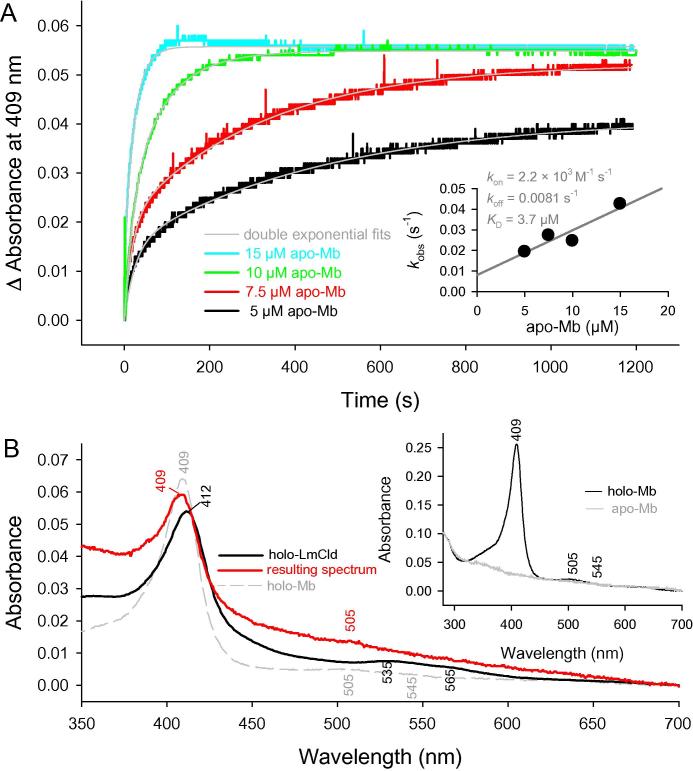
Heme transfer of LmCld to apomyoglobin. The transfer of the prosthetic group of 1 μM holo-LmCld to 5, 7.5, 10, and 15 μM apo-Mb was observed by the increase in absorbance at 409 nm. (A) Time traces when 1 μM holo-LmCld was mixed with 5–15 μM apo-LmCld in 100 mM phosphate buffer, pH 7.0. Double exponential fits are depicted in gray. The inset shows the linear dependence of *k*_obs1_ values from the apo-Mb concentration. The apparent association rate constant, *k*_on_, was calculated from the slope and the apparent dissociation rate constant, *k*_off_, from the intercept. (B) UV–vis spectra of undiluted holo-LmCld (black) prior to the measurement and one representative spectrum of the mixture of LmCld and Mb after 1200 s (red). Holo-Mb is depicted as gray dashed line for comparison. The inset depicts holo-Mb and apo-Mb after heme extraction. (For interpretation of the references to color in this figure legend, the reader is referred to the web version of this article.)

To probe if LmCld can act as a heme sensing protein, we performed the following experiment: we mixed an excess of apo-myoglobin (Apo-Mb) with holo-LmCld to see if apo-Mb can bind heme from holo-LmCld due do its higher binding affinity to the cofactor. The binding of heme to Mb can be followed by the increase of absorbance at 409 nm, the Soret maximum of holo-Mb, due to a different extinction coefficient of the heme group in its respective environments (Fig. 7A). The binding of heme to apo-Mb was dependent on the concentration of the latter. Time traces could be fitted double exponentially (below 10-times excess of apo-Mb) and single exponentially (above 10-times excess of apo-Mb). The plot of *k*_obs1_ versus apo-Mb concentration (Fig. 7A, inset) allows the calculation of the binding constants *k*_on_ (2.2 × 10^3^ M^−1^ s^−1^), *k*_off_ (0.0081 s^−1^), and a *K*_D_-value (3.7 μM). Spectroscopically the transfer of heme from LmCld to Mb is observed by a 3 nm blue-shift of the Soret maximum to 409 nm that is characteristic for holo-Mb (Fig. 7B).

### Enzymatic activity and cyanide binding

**Fig. 8:**
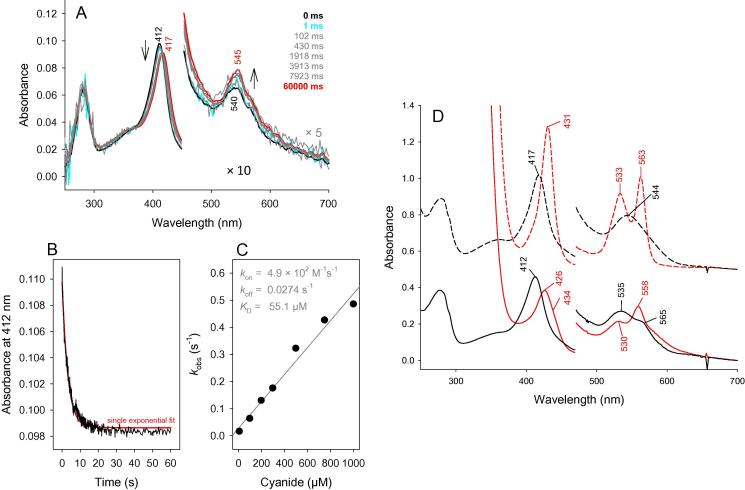
Cyanide binding to LmCld. Transient-state kinetics of binding of cyanide to holo-LmCld. (A) Spectral changes upon reaction of 3 μM ferric holo-LmCld with 1 mM cyanide measured in the conventional stopped-flow mode. (B) Typical time trace at 412 nm with a single-exponential fit. (C) Linear dependence of *k*_obs_ values from the cyanide concentration. (D) UV–vis spectra of 8 μM ferric (black solid line), ferrous (red solid line), cyanide-bound ferric (black dotted line) and cyanide-bound ferrous (red dotted line) of holo-LmCld. Conditions: 100 mM phosphate buffer, pH 7.0; 10 mM sodium dithionite (freshly prepared). (For interpretation of the references to color in this figure legend, the reader is referred to the web version of this article.)

The maximum *R*_Z_-value (*A*_Soret_/*A*_280_) of holo-LmCld was 1.1, reflecting a close to 50% heme occupancy. Holo-LmCld shows a Soret maximum (412 nm) and Q-bands (535, 565 nm) indicative of an N-ligated 6-coordinated low-spin (6cLS) heme [30] species that can be chemically reduced. Further it was possible to form a 6-coordinated low-spin heme-cyanide complex, which had its Soret band maximum at 417 nm (Fig. 8D). Holo-LmCld did not show any substrate concentration dependent catalase, chlorite dismutase, or peroxidase activity (data not shown).

Kinetics of cyanide binding were determined by conventional stopped-flow spectroscopy to assess the heme accessibility of holo-LmCld. Fig. 8A shows a representative spectral conversion of ferric holo-LmCld when being mixed with cyanide. Cyanide replaces an eventual distal high- or low-spin ligand to form a 6cLS species (*S* = 1/2), thereby shifting the Soret maximum from 412 to 417 nm. Ligand binding followed by stopped-flow spectroscopy at 412 nm was monophasic and *k*_obs_ values could be obtained from single-exponential fits (Fig. 8B). The apparent second-order rate constant for cyanide binding (*k*_on_ = 4.9 ± 0.4 × 10^2^ M^−1^ s^−1^) was calculated from the slope of the linear plot of *k*_obs_ versus cyanide concentration (Fig. 8C). The apparent dissociation rate constant (*k*_off_ = 0.03 ± 0.01 s^−1^) was determined from the intercept of the linear plots, allowing the calculation of the dissociation constant (*K*_D_ = 55.1 ± 23.5 μM) of the cyanide complexes from *k*_off_/*k*_on_ ratios.

## Discussion

Currently (October, 2014), almost 7500 sequences are found in the InterPro database of EMBL-EBI (http://www.ebi.ac.uk/interpro) when “chlorite dismutase” is used as a search template. More than 7000 of those sequences are predicted to have a similar fold and are part of the peroxidase-chlorite dismutase superfamily [Zámocký et al. (2015), this issue], formerly called the CDE structural superfamily [2]. Some of the sequences are experimentally confirmed chlorite degrading heme *b*-dependent oxidoreductases that are phylogenetically distinct and are classified as Clade I and Clade II Clds [2,3,22]. The majority of the proteins found via data base search is lacking the catalytic distal arginine and is therefore predicted to be incapable of converting chlorite to chloride and dioxygen and are thus referred to as Cld-like proteins. The true physiological role or even any other purpose or activity of these proteins remained elusive until very recently. Recent work by Dailey and co-workers [9] and Mayfield and co-workers [10] demonstrates that Cld-like proteins of Gram-positive bacteria play a role in heme biosynthesis. Thus, these Cld-like proteins were renamed into HemQ. The actual mechanism and interaction of HemQ with the other proteins involved in heme biosynthesis is not yet described, but it was hypothesized that HemQ has a regulatory role in the late stage of heme biosynthesis [9,10]. As such, HemQ should be able to bind and release the heme co-factor according to the needs of the organism.

Putative different oligomeric states of apo- (aggregated protein of approximately 1200 kDa) and holo-Cld-like protein (hexameric protein of approximately 187 kDa) from *S. aureus* (SaCld) were hypothesized to be the driving force of regulatory mechanisms in heme biosynthesis [10]. In contrast to SaCld, both apo- and holo-LmCld form hexameric assemblies (Fig. 1), but with the interesting observation that the *R*_Z_-values of small fractions of holo-LmCld, with higher oligomeric assemblies, were larger, indicating more stable cofactor incorporation. While LmCld forms hexamers in solution, it crystallizes as a pentamer. Also for functional chlorite dismutases there are reports of different oligomerization states. While chlorite degrading chlorite dismutases from Clade II form dimers [2,7,11], Cld from *Azospira oryzae* crystallizes as a hexamer but appears to be a pentamer in solution [31]. All other investigated Clds and Cld-like proteins crystallize as pentamers [12,22,23] or are reported to be tetramers in solution [6,32–35]. Therefore it seems that the oligomerization of Clds and Cld-like proteins is variable and might play a role for the physiological functions.

The crystal structure of apo-LmCld has a high degree of similarity of its overall fold to recently solved structures of Clade I Clds (Figs. 2 and S2). The most striking difference is a flexible stretch of amino acids (V105–K141, LmCld numbering) that is longer than in Clade I and Clade II chlorite dismutases. In chlorite degrading Clds, this flexible α-helical stretch of amino acids (V104–R131, NdCld numbering) forms the bottom of a wide open substrate channel. On the other hand, in LmCld and in other structures of Cld-like proteins [12], this flexible α-helical stretch forms a roof that covers partially the channel to the putative heme binding site. Nevertheless, LmCld was shown to bind hemin cooperatively (Fig. 3). Thus it can be concluded that this loop is flexible and can open and close the channel to the heme binding site.

Once heme has bound to LmCld, it can bind cyanide. The apparent binding rate constant of this low-spin ligand is very low, which is reflected by a hindered access to the active site, as shown by the accessibility calculations of the apo-LmCLd crystal structure. (Figs. 2 and S2). Nevertheless, binding of small cyanide to holo-LmCld is observed, suggesting that any other potential ligand might act as an activator, inhibitor or stabilizer of heme binding and dissociation. Therefore ligands might be involved in the regulatory mechanism of heme biosynthesis in *L. monocytogenes*.

Recent studies on chlorite dismutase-like proteins (HemQs) revealed their importance in heme biosynthesis of Gram-positive bacteria [9,10]. *ΔhemQ* strains of *S. aureus* cause a small colony (<1 mm) phenotype and the accumulation of coproporphyrinogen III under aerobic conditions. Still, it was ruled out that HemQ in *S. aureus* acts as an alternative coproporphyrinogen oxidase (CPO) that uses coproporphyrinogen III as a substrate to build protoporphyrinogen IX [10]. Since CPO, protoporphyrinogen oxidase (PPO), and ferrochelatase (Fc) form a functional complex and the loss of any of the three results in an apparent CPO deficiency, HemQ is hypothesized to be part of this functional complex performing an indirect regulatory task [10]. Heme biosynthesis might be turned on and off, depending whether heme is bound to HemQ or not. A similar mechanism could occur in *L. monocytogenes.*

Binding of hemin to apo-LmCld revealed *K*_D_ values of 7.2 μM (ECD) and 16.8 μM (ITC), which were similar to *K*_D_ values from HemQ of *M. tuberculosis* (30–40 μM) [9] and HemQ of *S. aureus* (1 μM) [10], respectively, and showed clear cooperativity (*n* > 1) (Fig. 3). Due to the cooperativity and the possibility to activate or inhibit heme binding by very small changes of the physiological conditions, such a regulatory mechanism is supported.

Spectroscopic studies of holo-LmCld clearly suggest that heme incorporation is most stable at neutral pH. At alkaline pH a limited amount of heme is released, whereas in acidic (<pH 5.5) conditions heme is released because the protein denatures (Fig. 4). This acidic instability of the holo-protein is even more pronounced at high salt conditions, where the decreased *R*_Z_-value indicates denatured protein at even higher pH (<pH 6.0) (Fig. 4A, inset). These data clearly show that heme binding in LmCld is fragile and is absolutely dependent on the environment.

The overall stability of holo- and apo-LmCld is high at neutral and alkaline conditions, with holo-LmCld being slightly more stable. Even at high temperatures, ECD spectra of LmCld show mainly intact secondary structural elements that differ from very uncommon minima of SaCld in the far UV region [10]. Refolding of the protein can be observed when performing a rescan after cooling down the protein solution. At temperatures larger than 70 °C, apo- and holo-LmClds aggregate but stay soluble (Fig. 5). The transitions observed in DSC thermograms at temperatures lower than 70 °C can be assigned to subtle rearrangements of secondary structural elements, that allow, for example, the release of heme (at around 50 °C) (Fig. 5E). These characteristics of stability and rearrangements of secondary structural elements are a prerequisite for LmCld to potentially act as a regulatory heme binding and releasing protein in heme biosynthesis of *Listeria*.

The ability of holo-LmCld to reversibly bind and release heme is shown in Fig. 6, where temperature was the driving force of heme dissociation and re-binding. While temperature is very unlikely the driving force under physiological conditions, this still proves that LmCld is equipped to regulate heme binding processes. Simply the presence of excess apo-myoglobin is sufficient to release heme from holo-LmCld and transfer it to form holo-Mb in a biphasic manner, depending on the apo-Mb concentration (Fig. 7). This allowed a calculation of a *K*_D_ value of 3.7 ± 2.9 μM that is clearly lower than the affinity of hemin to apo-LmCld. LmCld is therefore capable to act as a storage unit for heme until it is needed by another apo-protein with a higher affinity for the cofactor. This sheds light on the possibility that under physiological conditions the presence of another apo-heme-binding protein is sufficient to extract heme from LmCld and thus turns heme biosynthesis on and off. For SaCld, Mayfield and co-workers stated that in the presence of iron being available for ferrochelatase (Fc), HemQ is expected to have the heme bound (fed periodically by Fc). This status allows PPO and Fc to continuously produce protoporphyrin IX (PPIX) and heme, respectively [10]. In contrast, we propose an alternative regulatory role for HemQ in heme biosynthesis of Gram-positive bacteria, based on the presented data. The high *K*_D_ value (for hemin), the cooperativity in heme binding, and the ability to act as a storage unit for heme suggests that heme biosynthesis is triggered when LmCld (HemQ) is in its apo-form. In this scheme heme would be supplied from Fc to all other heme proteins in *Listeria* and, due to the high *K*_D_ value, heme would bind to LmCld last. When LmCld is in its holo-form, heme biosynthesis would be stopped. Upon decreasing heme concentrations, apo-heme proteins in *Listeria* can be supplied with heme by extracting it from holo-LmCld (HemQ), thereby transforming it into its apo-form and triggering heme biosynthesis again.

While the exact mechanism of regulation of Cld-like proteins in heme biosynthesis of Gram-positive bacteria still remains unknown, data presented in this work clearly show that LmCld is a potential HemQ, equipped with the necessary heme binding and releasing properties as well as a high stability to act as a regulatory protein in heme biosynthesis of this pathogenic microorganism. Considering this key physiological role and the absence of HemQ and other Cld-like proteins from animal genomes, HemQ could be an interesting target for antibiotics directed specifically against *Listeria* and other Gram-positive pathogens such as *S. aureus*.

## Figures and Tables

**Table 1:** Data collection and refinement statistics.

	LmCld (4WWS)
Wavelength (Å)	0.91841
Resolution range (Å)	48.76–2.0 (2.071–2.0)
Space group	P 1 21 1
Unit cell	78 128.32 78.15 90 105.91 90
Total reflections	415,140 (42,061)
Unique reflections	99,373 (9864)
Multiplicity	4.2 (4.3)
Completeness (%)	99.80 (99.99)
Mean *I*/*σ*(*I*)	6.77 (1.59)
Wilson B-factor	26.662
*R*-merge	0.196 (0.9392)
*R*-meas	0.2261
CC1/2	0.981 (0.563)
CC^∗^	0.995 (0.849)
Resolution *I*/*σ* = 2	2.08
*R*/free @ *I*/*σ*(*I*) > 2	0.2251
*R*-work	0.1975
*R*-free	0.2290
Number of non-hydrogen atoms	10,785
Macromolecules	9760
Ligands	5
Water	1020
Protein residues	1198
RMS(bonds)	0.003
RMS(angles)	0.59
Ramachandran favored (%)	99
Ramachandran outliers (%)	0.085
Clashscore	1.04
Average B-factor	24.90
Macromolecules	24.40
Ligands	21.80
Solvent	30.30

Statistics for the highest-resolution shell are shown in parentheses.

## Appendix A Supplementary data

Supplementary data 1 This document contains Supplementary Figs. S1 and S2.

## Footnotes

1 *Abbreviations used:* Cld, chlorite dismutase; LmCld, chlorite dismutase-like protein from *Listeria monocytogenes*; NwCld, chlorite dismutase from *Nitrobacter winogradskyi*; NdCld, chlorite dismutase from “*Candidatus* Nitrospira defluvii”; SaCld, chlorite-dismutase-like protein from *Staphylococcus aureus*; DyP, dye-decolorizing peroxidase; Mb, myoglobin; CPO, coproporphyrinogen oxidase; PPO, protoporphyrinogen oxidase; Fc, ferrochelatase; *R*_Z_, *Reinheitszahl*; MALS, multi-angle light scattering; ITC, isothermal titration calorimetry; DSC, differential scanning calorimetry; ECD, electronic circular dichroism; PPIX, protoporphyrin IX; ABTS, 2,2′-azino-bis(3-ethylbenzothiazoline-6-sulphonic acid).
