# Supplementary material for: Structure and heme-binding properties of HemQ (chlorite dismutase-like protein) from Listeria monocytogenes
Source: Arch Biochem Biophys. 2015 May 15;574:36–48. doi: 10.1016/j.abb.2015.01.010 (PMC4420033; doi:10.1016/j.abb.2015.01.010)

Figure S1. Structural environment of potential heme binding site of Cld-like proteins from *Listeria monocytogenes* (pdb-code: XXXX), *Geobacillus stearothermophilus* (pdb-code: 1T0T), *Thermoplasma acidophilum* (pdb-code: 3DTZ), and *Thermus thermophilus* (pdb-code: 1VDH). All structures are deposited as heme free apo-structures. The depicted prosthetic group is aligned from the structure of the functional Cld from ”*Candidatus* Nitrospira defluvii” (pdb-code: 3NN1).

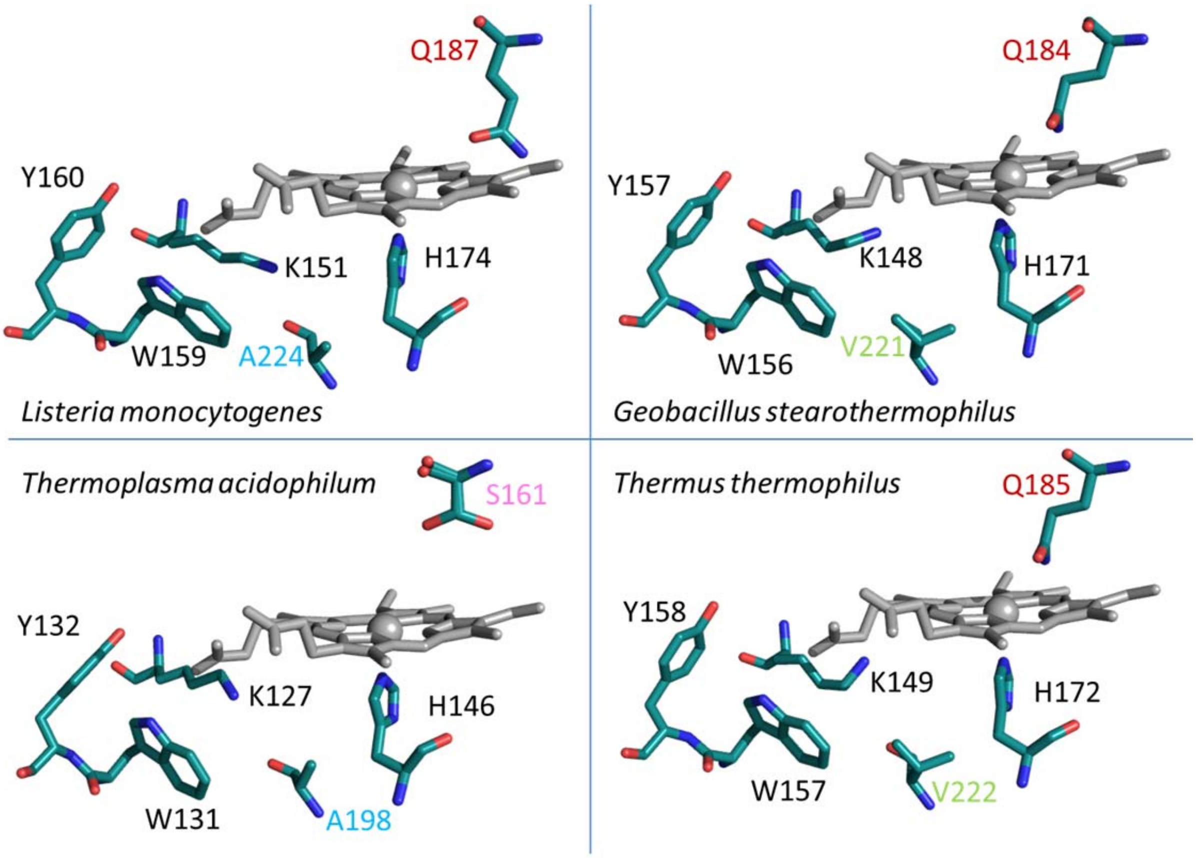

Figure S2. Accessibility of the heme binding cavity in chlorite dismutase-like protein from *Listeria monocytogenes* (A) and in chlorite dismutase from "*Candidatus* Nitrospira defluvii" (B). The proteins are depicted in ribbon representation with semitransparent surface, the two most important access channels to the heme binding site were determined with CAVER and are depicted as blue and green spheres. The putative heme (in LmCld) and the heme in NdCld are depicted as sticks. Figures were generated using PyMOL (http://www.pymol.org).

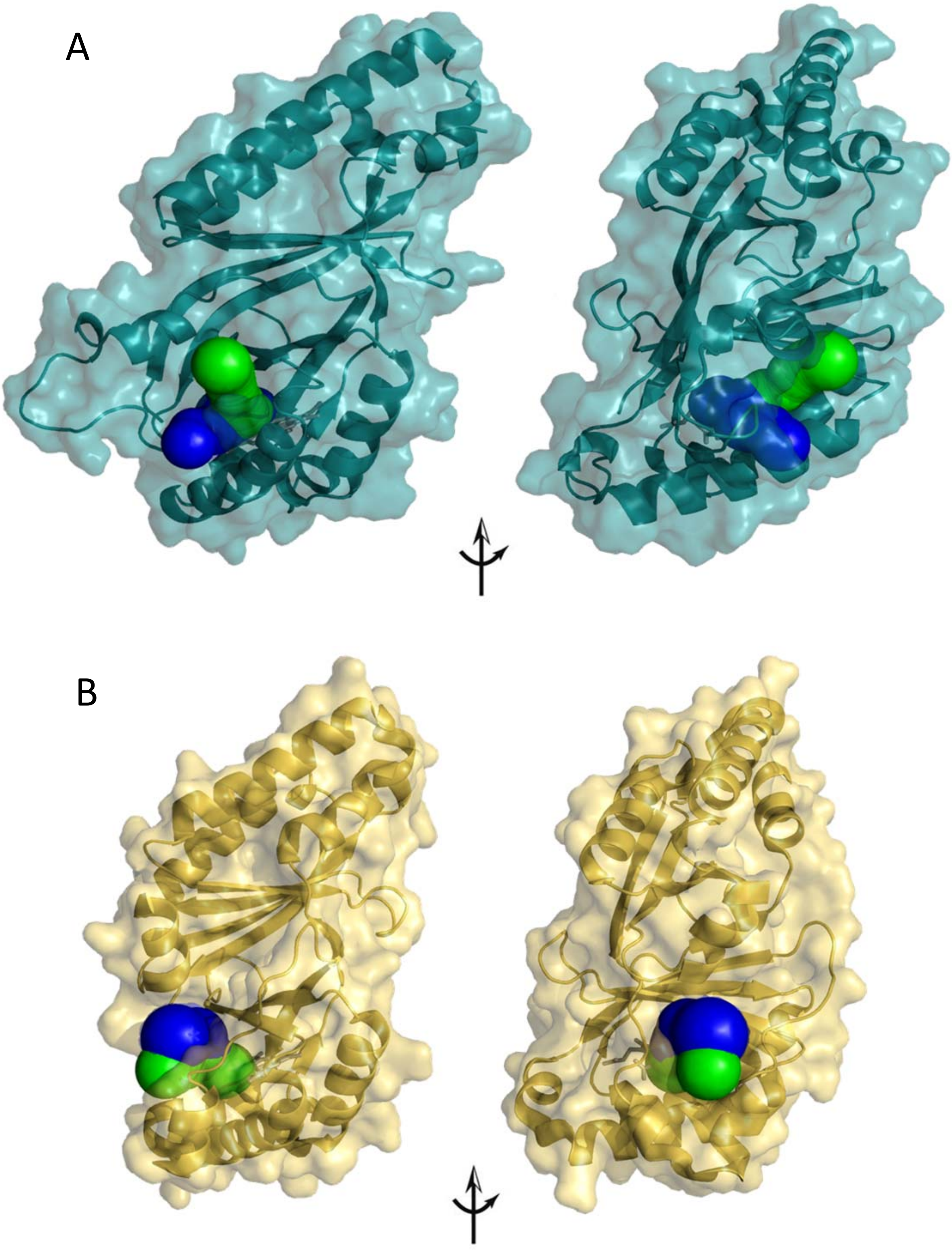

Supplement: Supplementary data 1 — This document contains Supplementary Figs. S1 and S2. [file mmc1.pdf]
